# Multiple schwannomas of the digital nerves and common palmar digital nerves: An unusual case report of multiple schwannomas in one hand

**DOI:** 10.1097/MD.0000000000014605

**Published:** 2019-03-08

**Authors:** Shuai Jiang, Hui Shen, Hui Lu

**Affiliations:** Department of Hand Surgery, The First Affiliated Hospital, School of Medicine, Zhejiang University, Hangzhou, Zhejiang Province, PR China.

**Keywords:** histopathology, MRI, multiple schwannomas, surgery

## Abstract

**Rationale:**

Benign schwannoma is the most common tumor of peripheral nerves while plexiform multiple schwannomas are rare conditions. This manuscript reported a case of multiple Schwannomas characterized by unusual location.

**Patient concerns:**

We report a 34-year-old workman presented with multiple nodules in his forefinger, middle finger and palm respectively for 5 years. He felt pain combined with paraesthesias in fingers and palm. The symptoms could be eased through rest and drugs medication.

**Diagnosis:**

Three Schwannomas were found in surgery respectively. Histologic findings confirmed the diagnosis of schwannomas.

**Interventions:**

We removed all of the 3 schwannomas completely without damaging the continuity of the nerve.

**Outcomes:**

Six months after the surgery, the patient had had not experienced any symptom recurrence.

**Lessons:**

Successful treatment of multiple schwannomas depends upon accurate diagnosis. Early surgery can improve the treatment outcome of multiple schwannomas.

## 1 Introduction

Schwannomas, also known as neurilemmomas, are the most frequently arising benign tumors involving peripheral nerve.^[1]^ They manifest approximately 5% of all benign soft tissue neoplasms, and their incidence in the upper limb ranges from 3% to 19% of all neurilemmomas.^[2]^ Schwannomas are the most common in 3rd and 6th decades with no sex predilection. They are usually solitary, slow growing, encapsulated neoplasm arising from the schwann cells of the myelin sheaths.^[3]^

Schwannomas are generally represented as painless swellings for several years before diagnosed which lead to difficulties in clinical diagnosis and treatment. In the upper limb, they may be mistaken for ganglion or neurofibroma. Pain, paraesthesia, or other symptoms may occur when the tumors reach sufficient size to compress the involved nerve. However, it is unusual for a schwannoma to exceed 3 cm in diameter. MRI and ultrasonography (USG) are helpful in the diagnosis and localization of these tumors. Preoperative accurate diagnosis and careful plan can be used to avoid unnecessary resection of the significant nerve and optimum efficiency nerve recovery. Surgical removal is usually curative. Single schwannoma is the most common tumor of all peripheral nerves, however, multiple schwannomas of different nerves in the same upper extremity are rare. We report a case of multiple neurilemmomas of the common palmar digital nerves and two individual digital nerves in one hand. Only few cases have been reported in the literature. We reviewed the literature and discussed the clinical features and therapeutic options in this report.

## 2 Case presentation

**Figure 1:**
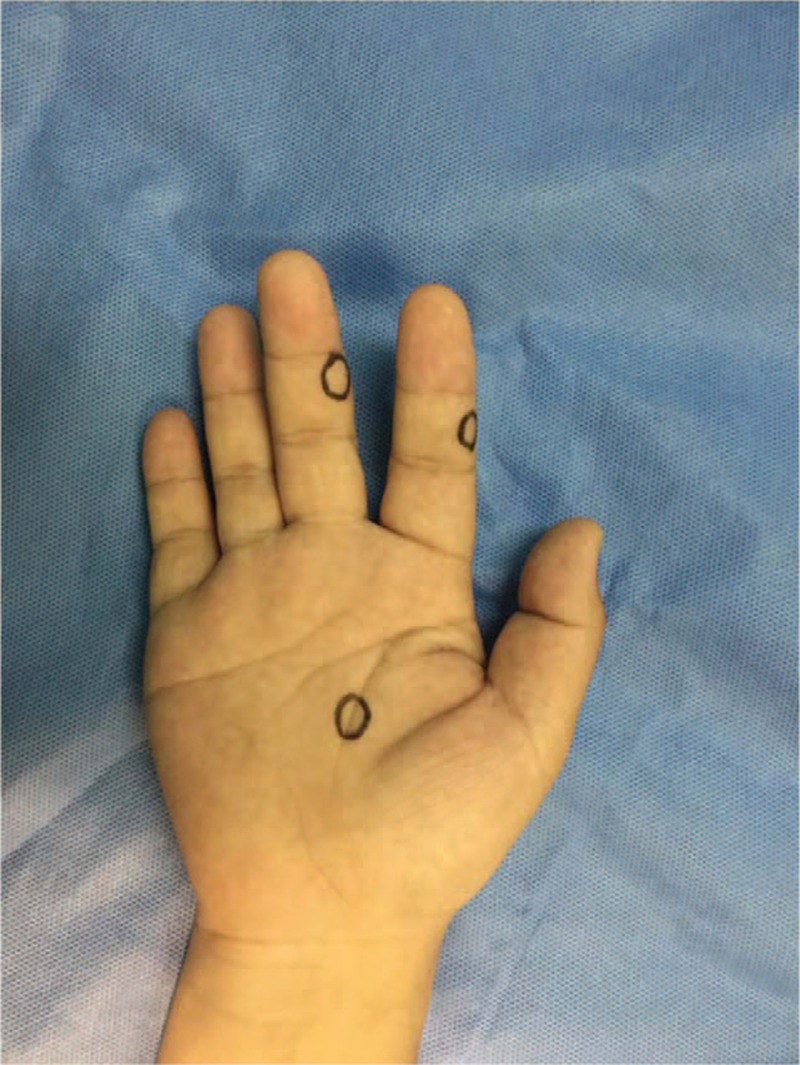
Surface location of the swellings in the middle phalanx of the index, middle fingers and the volar of third metacarpal respectively.

**Figure 2:**
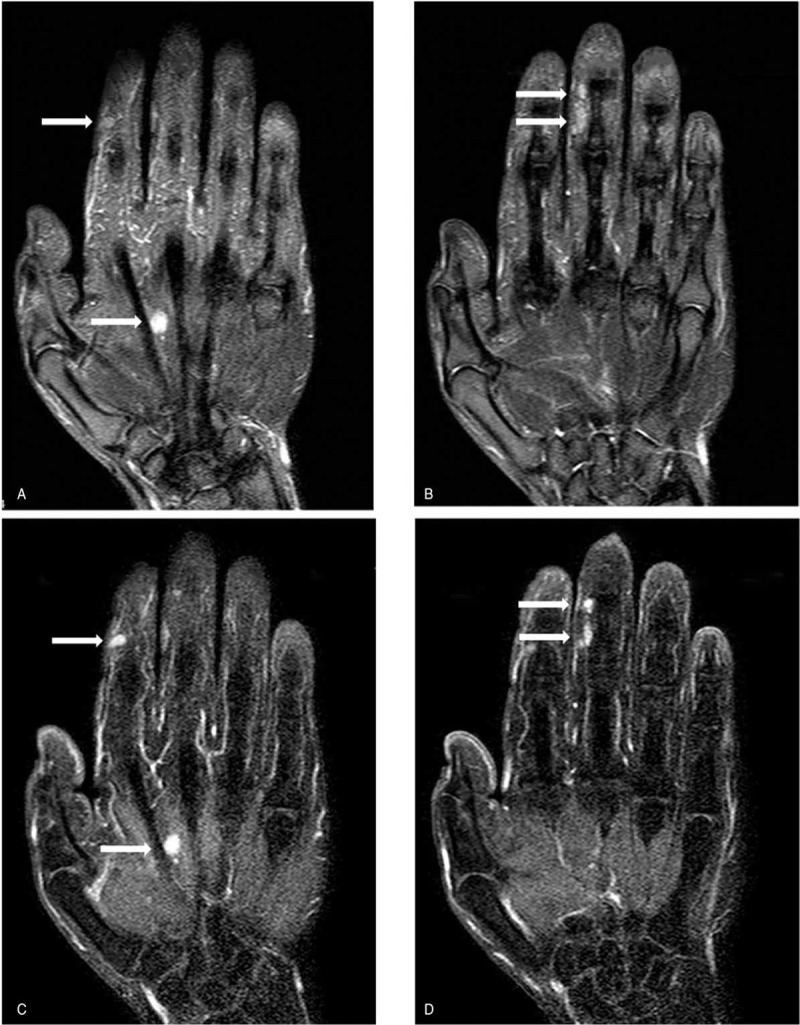
Coronal MR images showing round lesions (A-B): T2-weighted multi echo data image combination sequence (TR/TE: 2500/60) demonstrated four nodules of high intensity signal (arrows). (C-D): The tumors showed significant enhancement after administration of contrast agent (arrows).

A 34-year-old male labor worker presented with pain and paeresthesias on his right index, middle fingers and palm that had slowly increased in size over 5 years. The patient reported no preceding history of significant trauma or inflammation of the right hand. He complained of paeresthesias and pain in the area of the mass in the index, middle fingers and palm. These symptoms began approximately 2 years earlier. The pain got increased when pressure was applied on the nodules and with finger movements. The patient had no loss of sensation and he had the normal function of grip initially. And through rest and Non-Steroidal Anti-inflammatory Drugs medication (NSAIDs, 200 milligrams of Celebrex, twice a day), the patient's symptoms had been eased. On clinical examination, there were palpable, tender swellings in the middle phalanx of the index, middle fingers and the volar of third metacarpal respectively (Fig. 1). There was no discoloration of skin and the nodules had no discharge or bleeding since presentation. Local pain was triggered by applying pressure on the nodules. Movement at distal interphalangeal of the second and third finger were slightly limited because of pain. Tinel-Hoffman sign was positive on percussion of the nodules, accompanied by paraesthesias in the fingerpad of the fingers. Comparing to the opposite side, superficial sensation, as well as, static and dynamic sensory discrimination, was unaltered in the palm and fingerpad of the second and third fingers. Neither muscle atrophy nor impaired digital blood flow in the patient's hand was observed. Tumor biological markers and laboratory analyses including complete blood count, C-reactive protein, and electrolytes were normal. Radiographs of hand were normal. MRI (Fig. 2) examination (Siemes Essenza 1.5T) showed a mass in the middle of the second and third metacarpal, and masses in the radial side of index, middle fingers’ middle phalanx and the volar of third metacarpal. The mass showed low signal intensity on T1-weighted images and high signal intensity on T2-weighted images. The tumors showed significant enhancement after administration of contrast agent. The biopsy procedure was not to be performed in order to avoid wound contamination. The tentative diagnosis of neurinoma was made with a differential diagnosis of angioma.

### 2.1 Therapeutic intervention

**Figure 3:**
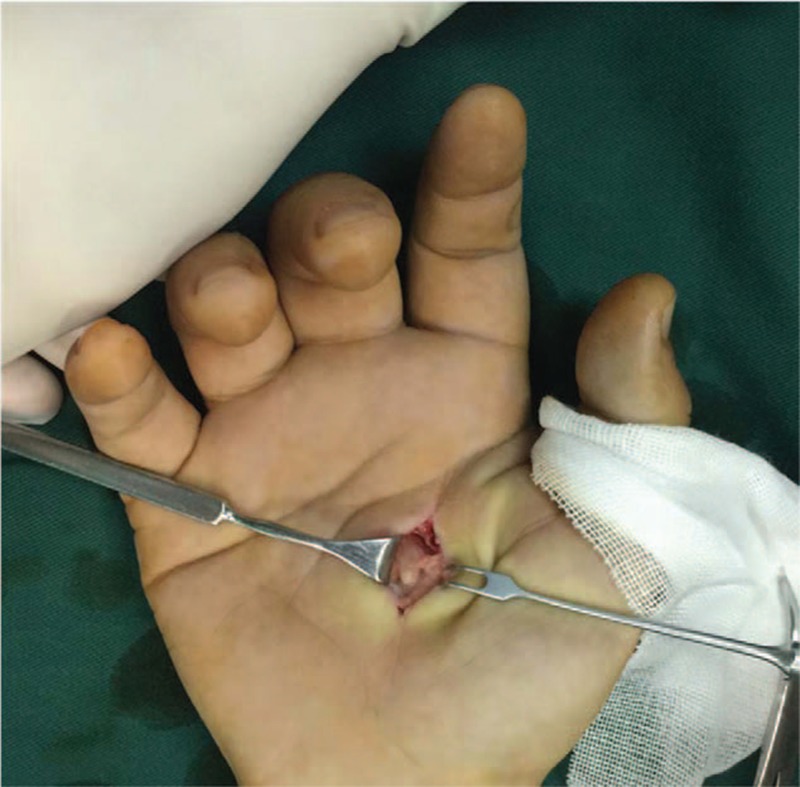
A 12 mm × 7 mm × 4 mm yellowish tan, firm mass was separated from common palmar digital nerves of median nerve.

**Figure 4:**
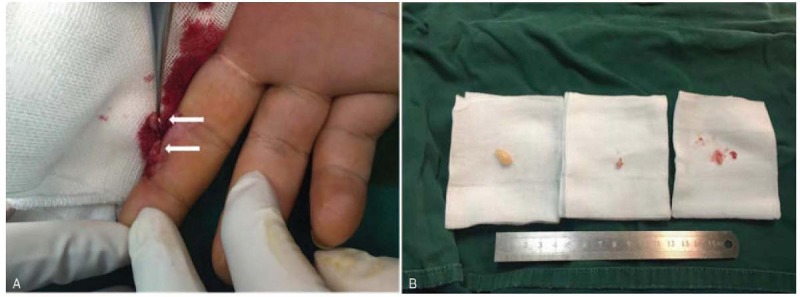
(A) Two nodules located on the radial side at the level of the middle phalanx in middle finger; (B) Four tumors in 3 locations respectively in the index finger, middle finger and palm were removed completely.

The surgical procedure was performed under brachial plexus block. A brachial tourniquet was used and the procedure was carried out with surgical loupes. With volar approach, a longitudinal incision was made on the middle of the second and third metacarpal. A 12 mm × 7 mm × 4 mm yellowish tan, firm mass was separated from common palmar digital nerves of median nerve (Fig. 3). The nerve fascicles were splayed by the tumors and were preserved during the operation. In addition, a nodule located in the index digital nerve on the radical side at the level of the middle phalanx in index finger and another 2 nodules located on the radial side at the level of the distal interphalangeal joint in middle finger were removed. The nodules were 5, 6, and 4 mm in diameter respectively (Fig. 4). Surgical treatment was performed using a surgical loupe in order to avoid damaging the fascicular structure of nerves. All nodules were excised carefully without vast dissection of tissues of the fingers.

### 2.2 Histopathology examination

**Figure 5:**
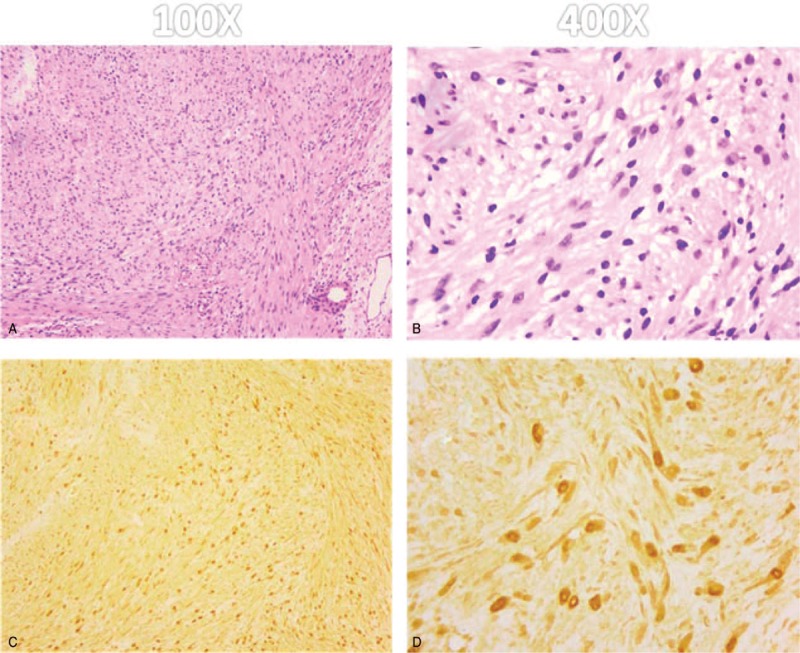
Photomicrograph of the tumor. (A-B): Microscopic specimen of the lesion shows long spindle-shaped tumor cells and nuclei palisading with multiple fascicles (Hematoxylin-eosin, H & E). (C-D): The tumor cells stain positive for S-100 protein and exhibit strong immunoreactivity for S-100 protein.

Grossly the tumor tissue appeared as soft, solitary, encapsulated with well-defined surface and had a yellowish color. Microscopically-well circumscribed tumor composed of spindle shaped cells arranged in a palisading fashion. There was no mitotic activity or malignancy features were seen. Immunostaining demonstrated strong extensive S-100 immunoreactivity of the nodules with CD34, Desmin, SMA, and EMA negativity (Fig. 5). These findings confirmed the diagnosis of Schwannomas.

### 2.3 Follow-up and outcomes

Postoperatively, there were no motor or sensory deficits. No infection was found after surgery. One week after the surgery, the patient was able to perform full motion of his operated hand. Symptoms of pain and paraesthesias resolved, and his finger range of motion recovered to the normal level. Tinel-Hoffman sign was negative. The patient was followed up by clinic every 3 months. Two years after the surgery, the patient had not experienced tumor recurrence.

## 3 Discussion

Schwannomas are typically benign slow growing nerve sheath tumors, which usually arise on the flexor aspect of the extremities. Generally, they present as solitary tumors, although there are reports of patients with multiple tumors in the literature. In material presented by Ogose et al, the incidence of multiple schwannomas had been reported as 4.6% of all patients diagnosed with schwannoma.^[4]^ The case we reported showed multiple localized schwannomas confined to three different nerves in one hand. To our knowledge, it is extremely rare in the literature.

Pain and swelling are the usual presentation in general clinical physical examination. The similar features sharing with other soft tissue tumors cause difficulties in diagnosis.^[5]^ Schwannoma may be misdiagnosed as neurofibroma, lipoma and ganglion preoperatively.^[6,7]^ Schwannoma can arise in association with any peripheral nerve but it generally arises in the sensory portion of the nerve. Neurological symptoms like pain, paraethesia and numbness are presented when the tumors compress the involved nerve.^[8,9]^ On clinical exam, the swelling can be mobile from side to side, but not in the longitudinal axis of the nerve. Percussion induces painful paraesthesiae in the area of the nerve of origin similar to Tinel sign.^[10]^ A full history and careful detailed examination can improve the preoperative diagnostic accuracy. In our case, symptoms such as palpable tumor mass, pain, paraesthesias, and positive Tinel-Hoffman sign enhanced the evidence to diagnosis schwannomas.

USG and MRI are potent for localizing the tumors in hand as they are noninvasive and easy to manipulate. Ultrasonography works as a first-line imaging examination to localize and evaluate lesions of the hand. Schwannoma is homogenous, hypoechoic mass which exhibit enhanced through transmission, target appearance, pseudocystic appearance on ultrasonography, which is rather similar to ganglion cysts.^[11,12]^ Therefore, schwannoma of the hand is hard to distinguish from a cystic lesion. Till now, MRI is still the most competent imaging modality used to localize the tumors and to plan for surgery.^[13]^ Schwannoma on MRI is revealed by high signal intensity on T2 scans and low signal intensity on T1 scans. Possibly, it can lead to misdiagnosis as nerve sheath tumors and ganglions that have same appearance with Schwannoma on MRI. However, we can identify schwannomas from ganglions through the administration of gadolinium in which ganglions do not show any enhancement. In addition, the location of the lump to the nerve is another key to separate them, where schwannomas is eccentric to the nerve while neurofibromas is central to the nerve.

Histology of schwannoma shows the typical features including areas of Antoni type A, which characterized as compact bundles of Schwann cells, or Antoni type B, difined as loose matrix of oval cells. Both of the 2 types and a distinct capsule could be seen in our case. Compared with neurofibromas, schwannomas shows greater cellularity in Antoni type A. The cellular areas of the schwannomas express positive S-100 protein strongly, while neurofibromas express variable S-100 protein.^[14]^ It is also a key characteristic to distinguish schwannomas from leiomyosarcoma and fibrosarcoma.

Surgery is the main therapeutic strategy for schwannoma, the principle of which is enucleation of tumor with no nerve damage.^[15]^ Proper microsurgical dissection is as important for success as in a bloodless surgery, such utilization of loupe or microscopical magnification is strongly advised to use in order to avoid the nerve fibers damage.^[16]^ It requires surgeons pay meticulous attention to preserve functionally important sensory and motor branches without making unnecessary sacrifice. To protect the nerve, we used surgical loupes to remove the schwannomas in our case, and ultimately no nerve damage was found. Successful treatment of multiple schwannomas depends upon accurate early diagnosis. Surgery can improve the treatment outcome of multiple schwannomas.

## Acknowledgment

First and foremost, I would like to show my deepest gratitude to my colleagues Dr Hui Lu and Dr Hui Shen who have provided me with valuable assist in every stage of writing this paper. Last but not least, I’ d like to thank all my friends, especially my lovely wife for her encouragement and support.

## Author contributions

**Data curation:** Shuai Jiang, Hui Shen.

**Funding acquisition:** Hui Lu, Shuai Jiang.

**Methodology:** Hui Shen.

**Writing – original draft:** Shuai Jiang.

**Writing – review & editing:** Shuai Jiang, Hui Lu.

Shuai Jiang orcid: 0000-0002-4401-6595.
